# The notch pathway promotes NF-κB activation through Asb2 in T cell acute lymphoblastic leukemia cells

**DOI:** 10.1186/s11658-018-0102-4

**Published:** 2018-08-09

**Authors:** Wei Wu, Li Nie, Li Zhang, Yan Li

**Affiliations:** 1 Department of Clinical Laboratory, Renmin Hospital of Wuhan University, Wuhan, 430060 People’s Republic of China; 2 Department of Geriatrics, Renmin Hospital of Wuhan University, Wuhan, 430060 People’s Republic of China; 3 Department of Hematology, Renmin Hospital of Wuhan University, Wuhan, 430060 People’s Republic of China

**Keywords:** Notch1, NF-κB, Asb2, T cell acute lymphoblastic leukemia

## Abstract

**Background:**

Oncogenic Notch1 is known to activate the NF-κB pathway in T cell acute lymphoblastic leukemia (T-ALL) and to up-regulate the transcription of Asb2α, a specificity factor for an E3 ubiquitin ligase complex that plays an important role in hematopoietic differentiation. Therefore, we hypothesize that Notch1 might regulate the NF-κB pathway through Asb2α.

**Methods:**

The study involved down-regulation of Notch1 in T-ALL cell lines (CCRF-CEM cells and MOLT-4 cells) through treatment with gamma-secretase inhibitor (GSI) as well as the modulation of Asb2 in CCRF-CEM cells and MOLT-4 cells through transduction with lentivirus carrying *Asb2* or *Asb2*-shRNA. Experiments using real-time PCR, western blot and co-immunoprecipitation were performed to evaluate the expression levels of related genes. Cell proliferation and apoptosis were measured while the expression of Asb2 was enhanced or inhibited.

**Results:**

Here, we demonstrated for the first time that Notch1 can activate the transcription of Asb2α, which then stimulates activation of NF-κB in T-ALL cells. Asb2α exerts its effects by inducing degradation and dissociation of IκBα from NF-κB in T-ALL cells. Moreover, specific suppression of Asb2α expression can promote apoptosis and inhibit proliferation of T-ALL cells.

**Conclusion:**

Notch1 modulates the NF-κB pathway through Asb2α, indicating that Asb2α inhibition is a promising option for targeted therapy against T-ALL.

## Background

The NF-κB protein functions as a transcription factor that mediates a broad range of biological processes, including cell survival, proliferation and differentiation [1]. In the canonical NF-κB pathway, the NF-κB protein is bound and inhibited by the IκB protein. Once a stimulus, such as a pro-inflammatory cytokine, activates the IκB kinase (IKK) complex, the IκB protein becomes phosphorylated, targeting it for ubiquitination and proteasomal degradation. The degradation of IκB leads to the release and nuclear translocation of NF-κB, thus inducing the transcriptional activation of its downstream genes, including IκBα [2]. The newly synthesized IκBα protein associates with NF-κB and rapidly shuts down the NF-κB response, ensuring that the expression of NF-κB-induced genes is transient [3].

Considering its role in cell survival and proliferation, abnormal or uncontrolled activation of NF-κB is frequently encountered in several lymphoid malignancies and solid tumors [4–7]. Therefore, NF-κB regulation has been intensely studied in the context of oncogenesis, and it represents a promising target for cancer therapy. Some research groups have revealed a link between Notch and NF-κB in human T-ALL and in a mouse model of T cell leukemia [8–10]. It is well established that abnormal activation of Notch signaling is able to augment the activation of NF-κB. However, the underlying mechanism remains unclear. Notch receptors act as membrane-tethered transcription factors. Upon the binding of its ligands, two successive proteolytic cleavages of the receptor occur to release the intracellular domain of Notch (ICN). The ICN then translocates into the nucleus to initiate the transcription of a number of target genes by interacting with its DNA-binding partner, CSL, and recruiting transcriptional co-activators such as mastermind [11–13]. Among the known downstream target genes of the Notch signaling pathway, the ankyrin repeat-containing protein with a suppressor of cytokine signaling box 2 (*Asb2*) gene is of particular interest due to its vital function in hematopoietic differentiation. Moreover, some studies have shown that Notch signaling initiates the degradation of Jak2, Jak3 and E2A proteins by up-regulating the expression of *Asb2* [14, 15]. As the specificity subunit of an E3 ubiquitin ligase complex, the classic function of the Asb2 protein is to target certain proteins for ubiquitination and degradation by the proteasome [16, 17]. The *Asb2* gene encodes two different isoforms, Asb2α and Asb2β, which are involved in hematopoietic differentiation and myogenic differentiation, respectively [16, 18]. Asb2α proteins were first identified in retinoic acid-induced acute promyelocytic leukemia (APL) cells [19]. Recently, expression of Asb2α was observed in normal hematopoietic cells, where it contributes to hematopoiesis [20, 21]. Considering these findings, we hypothesize that Notch signaling may influence NF-κB activity through the Asb2α protein in T-ALL cells.

In this report, we show that Notch signaling can up-regulate *Asb2* transcription and NF-κB activation in T-ALL cells. Inhibition of Asb2α expression can significantly decrease Notch-induced NF-κB activation, suggesting that Notch signaling mediates NF-κB activation through Asb2α. In addition, we explore the mechanism whereby Asb2α promotes NF-κB activation. Our results demonstrate that Asb2α is able to target IκBα for destruction and thus is able to free NF-κB from an inhibitory status. Our findings are the first to reveal that Asb2α is an important regulator between Notch and the NF-κB signaling pathway in T-ALL cells, indicating that Asb2α might play a vital role in T-ALL formation and shedding light on a therapeutic target for T-ALL disease.

## Methods

### Reagents

Roswell Park Memorial Institute (RPMI) 1640, Dulbecco’s modified Eagle’s medium (DMEM) and fetal bovine serum (FBS) were obtained from Invitrogen (Carlsbad, CA, USA). Propidium iodide was obtained from Sigma (Oakville, ON, Canada). FITC-conjugated annexin V was purchased from BD Biosciences (Mississauga, ON, Canada). The Cell Counting Kit-8 (CCK-8) was purchased from Beyotime Institute of Biotechnology (China). DMSO, GSI and MG132 were also purchased from Sigma (Oakville, ON, Canada).

### Cell culture and treatment

Human embryonic kidney (HEK) 293 cells were cultured in DMEM supplemented with 10% FBS. The CCRF-CEM human immature T cell line was obtained from Shanghai Bioleaf Biotech (Shanghai, China). The human leukemia T-cell line (MOLT-4 cells) was purchased from Procell (Wuhan, China). CCRF-CEM and MOLT-4 cells were cultured in RPMI 1640 medium supplemented with 10% FBS at 37 °C in a humidified atmosphere of 5% CO_2_ in air. For the chemical treatment experiments, exponentially grown CCRF-CEM cells and MOLT-4 cells were harvested, resuspended (at 4 × 10^5^ cells/ml) in fresh culture medium and incubated for 24 h before treatment with 5 μM MG132 or 10 μM GSI for 24 h. DMSO-treated cells served as the control. For viral infection experiments, exponentially grown CCRF-CEM cells and MOLT-4 cells were harvested, resuspended (at 1 × 10^5^ cells/ml) in fresh culture medium and incubated for 12 h before being infected with 4 × 10^6^ TU of lentivirus for 72 h.

### Vector construction

The sequences for the *Asb2* shRNA2 were as follows: sense 5’-CAGGCAGGCTGATTAGATATTCAAGAGATATCTAATCAGCCTGCCTGTTTTTTCTCGAGG-3’ and antisense 5’-GATCCCTCGAGAAAAAACAGGCAGGCTGATTAGATATCTCTTGAATATCTAATCAGCCTGC CTG-3’. Plasmids pLVX-shRNA2-m and PLVX-mcmv-ZsGreen1 were purchased from Biowit Technologies, Ltd. (China). pLVX-shRNA2-m was first digested with *Pst*I and then filled in with Klenow. The larger fragment was then extracted and cleaved with *BamH*I. Finally, the *Asb2* shRNA oligonucleotides were synthesized, annealed and ligated into the pLVX-shRNA2-m vector to obtain pLVX-shRNA2-hASB2. pCMV-ASB2-HA and Asb2 deletion constructs were kindly provided by Dr. Jay L. Hess (University of Michigan Medical School, Ann Arbor, MI, USA). The full-length HA-tagged hAsb2 sequence was then cloned into the pLVX-mcmv-ZsGreen1 vector through *EcoR*I and *Not*I digestion and ligation. The sequences for the *Asb2* shRNA1 were as follows: sense 5’-CACCCGAACATCGACGCCTATATTTCAAGACGATA TAGGCGTCGATGTTCG TTTTTTG-3′ and antisense 5’-AGCTCAAAAAACGAACATCGACGCCTATATCGTCTTGAAA TATAGGCGTCGATGTTCG-3’. The sequences for the *Asb2* shRNA3 were as follows: sense 5’-CACCGGCTGATTAGATACCTGAA TTCAAGACGTTCAGGTATCTAATCAGCCTTTTTTG-3’ and antisense 5’-AGCTC AAAAAAGGCTGATTAGATACCTGA ACGTCTTGAATTCAGGTATCTAATCAGCC-3’.

.

### Lentivirus packaging and production

The 293 T cell line was used to obtain lentivirus from packaging plasmids and the lentiviral vector. Approximately 24 h before transfection, 6–8 × 10^6^ 293 T cells were seeded in 10-cm tissue culture plates in 10 ml of growth medium and then incubated at 37 °C with 5% CO_2_ overnight. The cells were 80–90% confluent at the time of transfection. Approximately 2–4 h before transfection, the medium was replaced with 5 ml of fresh complete growth medium. The 293 T cells were transfected with a highly efficient transfection reagent (Biowit Technologies, Ltd.) according to the manufacturer’s instructions. Approximately 12–16 h after transfection, the transfection medium was replaced with 10 ml of fresh complete growth medium, and the cells were incubated at 37 °C for an additional 48 h. The cells were then harvested, and the lentiviral supernatant was filtered through a 0.45-μm low-protein-binding filter to remove cellular debris.

### Immunoprecipitation

HEK293 cells were lysed with Cell Lysis Buffer for Western and IP (Beyotime) at 4 °C for 15 min. The cell extracts were incubated with anti-HA antibody (1:5000) overnight at 4 °C. Agarose affinity beads were then added and incubated with the extracts for 1 h at room temperature. The beads were washed 3 times with RIPA buffer (1% Nonidet P-40, 0.5% sodium deoxycholate, and 0.1% sodium dodecyl sulfate [SDS] in phosphate-buffered saline [PBS]). Proteins were eluted by boiling in SDS loading buffer, resolved by SDS-polyacrylamide gel electrophoresis (PAGE), and detected by Western blot. The primary antibody directed against the HA tag was obtained from Santa Cruz.

### Immunoblot analysis

The cells were lysed in Cell Lysis Buffer for Western and IP (Beyotime) and incubated at 4 °C for 15 min. Protein extracts were separated by SDS-PAGE and then transferred to polyvinylidene fluoride membranes before overnight incubation with primary antibodies directed against Asb2 (Santa Cruz), IκBα (Cell Signaling Technology, 1:600 dilution), NF-κB-p65 (BioWorld), Caspase 3 (Proteintech Group, 1:2000 dilution), NICD (abcam), Hes1 (abcam), lamin (Wuhan Boster Biological Technology) or GAPDH (Wuhan Boster Biological Technology) at 4 °C. The membrane was washed with 0.1% Tween-20 in Tris-buffered saline and then incubated with horseradish peroxidase-conjugated anti-rabbit or anti-goat IgG secondary antibody (Wuhan Boster Biological Technology) for 1 h at room temperature. The immunoreactive bands were visualized using an ECL Western blotting detection kit (Thermo, Waltham, MA, USA) with light-sensitive film.

### Real-time quantitative reverse-transcription PCR

RNA was extracted from cells using TRIzol reagent (Invitrogen), and cDNA was synthesized using a First Strand cDNA Synthesis Kit (Fermentas) according to the manufacturer’s instructions. Real-time quantitative reverse-transcription PCR (RT-qPCR) was performed using 2X SYBR Green/Fluorescein qPCR Master Mix (Fermentas) on an ABI 7900 Sequence Detection System (Applied Biosystems). The data were analyzed using the comparative CT method (ABI User Bulletin number 2). The primer sets were as follows: Asb2, forward 5’-CGTGGTGCAGTTCTGTGAGT-3′ and reverse 5’-GTGAGCCAGAGGTCTTGGAG-3’; IκBα, forward 5’-GCAAAATCCTGACCTGGTGT-3′ and reverse 5’-GCTCGTCCTCTGTGAACTCC-3’; Actin, forward 5’-AGCGAGCATCCCCCAAAGTT-3′ and reverse 5’-GGGCACGAAGGCTCATCATT-3’.

### Cell viability assay (CCK-8 assay)

Cell viability was determined using a CCK-8. Briefly, 5 × 10^3^ CCRF-CEM cells were resuspended in 100 μl of RPMI 1640 medium in a 96-well plate. Lentiviruses harboring empty vector, *Asb2* or *Asb2*-shRNA were then incubated with the cells for 72 h. After the cells were infected, 10 μl of CCK-8 solution was added to each well, and the 96-well plate was continuously incubated at 37 °C for 2.5 h. The OD value for each well was then read at 450 nm on a microplate reader (Multiskan, Thermo, USA) to determine cell viability. The assay was repeated three times. Cell viability was calculated as follows:

$$ \mathrm{Cell}\kern0.17em \mathrm{viability}\;\left(\%\right)=\frac{\mathrm{OD}\left(\mathrm{experiment}\right)\hbox{-} \mathrm{OD}\left(\mathrm{blank}\right)}{\mathrm{OD}\left(\mathrm{control}\right)\hbox{-} \mathrm{OD}\left(\mathrm{blank}\right)}\times 100 $$

### Analysis of T-ALL cell apoptosis

ALL cell apoptosis was detected using an annexin V/propidium iodide (PI) staining assay. Cells (1 × 10^6^) were washed in ice-cold PBS and resuspended in 500 μl of annexin V binding buffer (140 mM NaCl, 2.5 mM CaCl_2_, 1.5 mM MgCl_2_, and 10 mM HEPES, pH 7.4) containing annexin V-FITC and PI (1 μg/ml) before being incubated for 30 min at 4 °C. FACS analysis was performed with Cell Quest-Pro software, and cells negative for both annexin V and PI were considered viable.

### Statistical analyses

All the experiments were performed three times. The data are expressed as mean ± standard deviation (SD). Differences between the control and experimental results were tested by Student’s t-test (two-tailed) and one-way analysis of variance (ANOVA). All statistical analyses were carried out using SPSS version 22.0 (SPSS Inc., Chicago, IL, USA) and *p* < 0.05 was considered to indicate a significant difference.

## Results

### Notch signaling can initiate Asb2 transcription and NF-κB activation in T-ALL cells

**Fig. 1:**
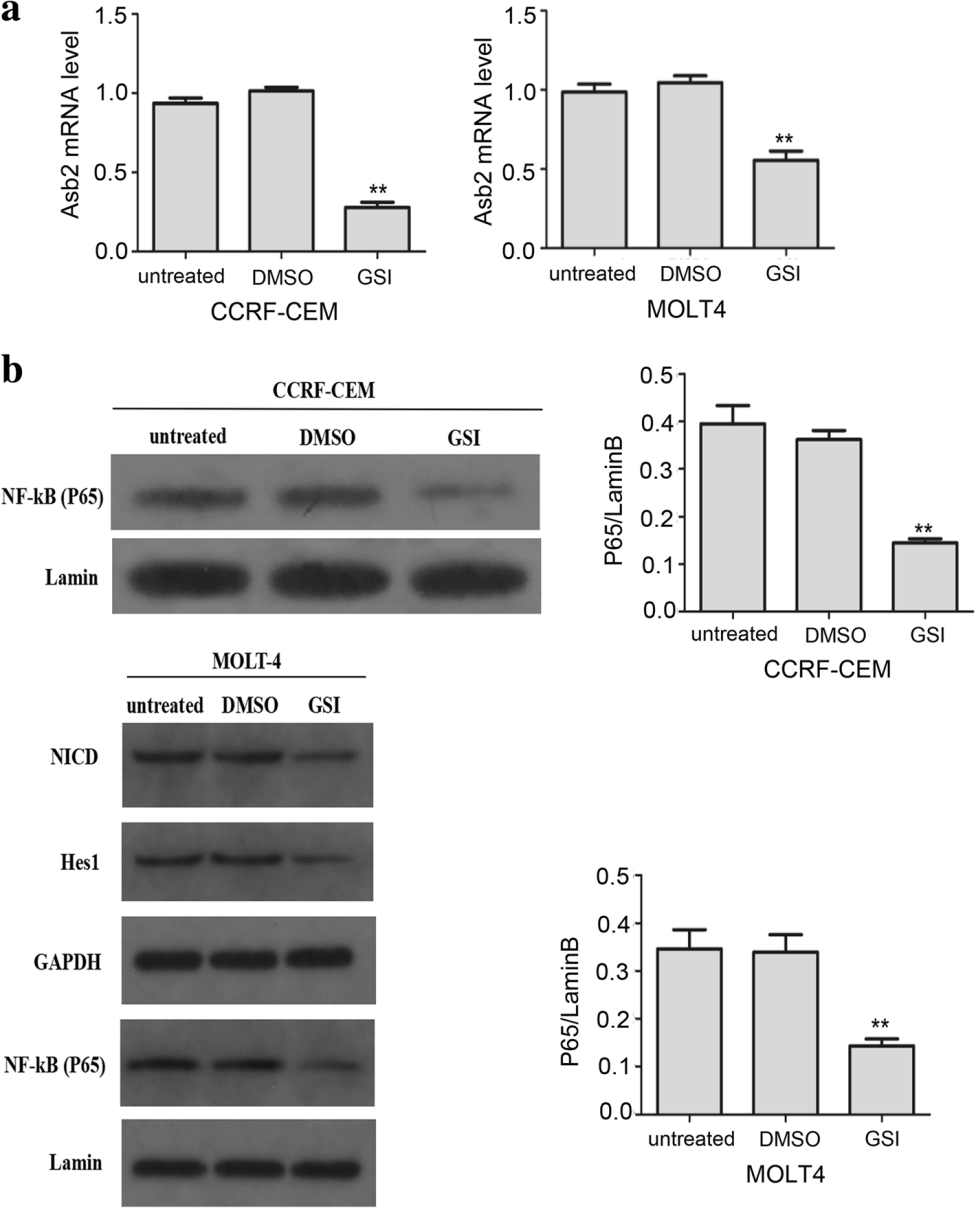
Notch signaling up-regulates *Asb2* transcription and NF-κB activation. CCRF-CEM cells and MOLT-4 cells were treated with DMSO (vehicle) or with 10 μM GSI for 24 h. Untreated cells served as the control. **a** Levels of *Asb2* mRNA as assessed by qRT-PCR. **b** Levels of NF-κB-p65 in the nuclei of CCRF-CEM cells and MOLT-4 cells. The data are presented as the means ± SD, **P < 0.01 versus DMSO control. Each assay was conducted in triplicate

Notch signaling can stimulate *Asb2* gene transcription in NIH 3 T3 cells (a mouse embryonic fibroblast cell line) and in lymphoid cells [14]. However, whether the same stimulation occurs in T-ALL cells remains unclear. To answer this question, a human T cell lymphoblast-like cell line (CCRF-CEM) and a human leukemia T-cell line (MOLT-4) were treated with gamma-secretase inhibitor (GSI), which is a Notch-specific inhibitor. *Asb2* transcription was significantly decreased 24 h after GSI treatment of CCRF-CEM cells and MOLT-4 cells (Fig. 1a). This result suggests that activated Notch signaling can up-regulate *Asb2* transcription. Notch signaling is known to be constitutively activated, thus inducing NF-κB activation in T-ALL cells [8–10]. To confirm this constitutive behavior, we measured the nuclear NF-κB levels in CCRF-CEM cells and MOLT-4 cells 24 h after DMSO or GSI treatment. GSI treatment significantly decreased the amount of NF-κB in the nuclei of CCRF-CEM cells and MOLT-4 cells, in contrast to DMSO treatment (Fig. 1b).

### Notch signaling regulates NF-κB activation by activating Asb2 transcription in T-ALL cells

**Fig. 2:**
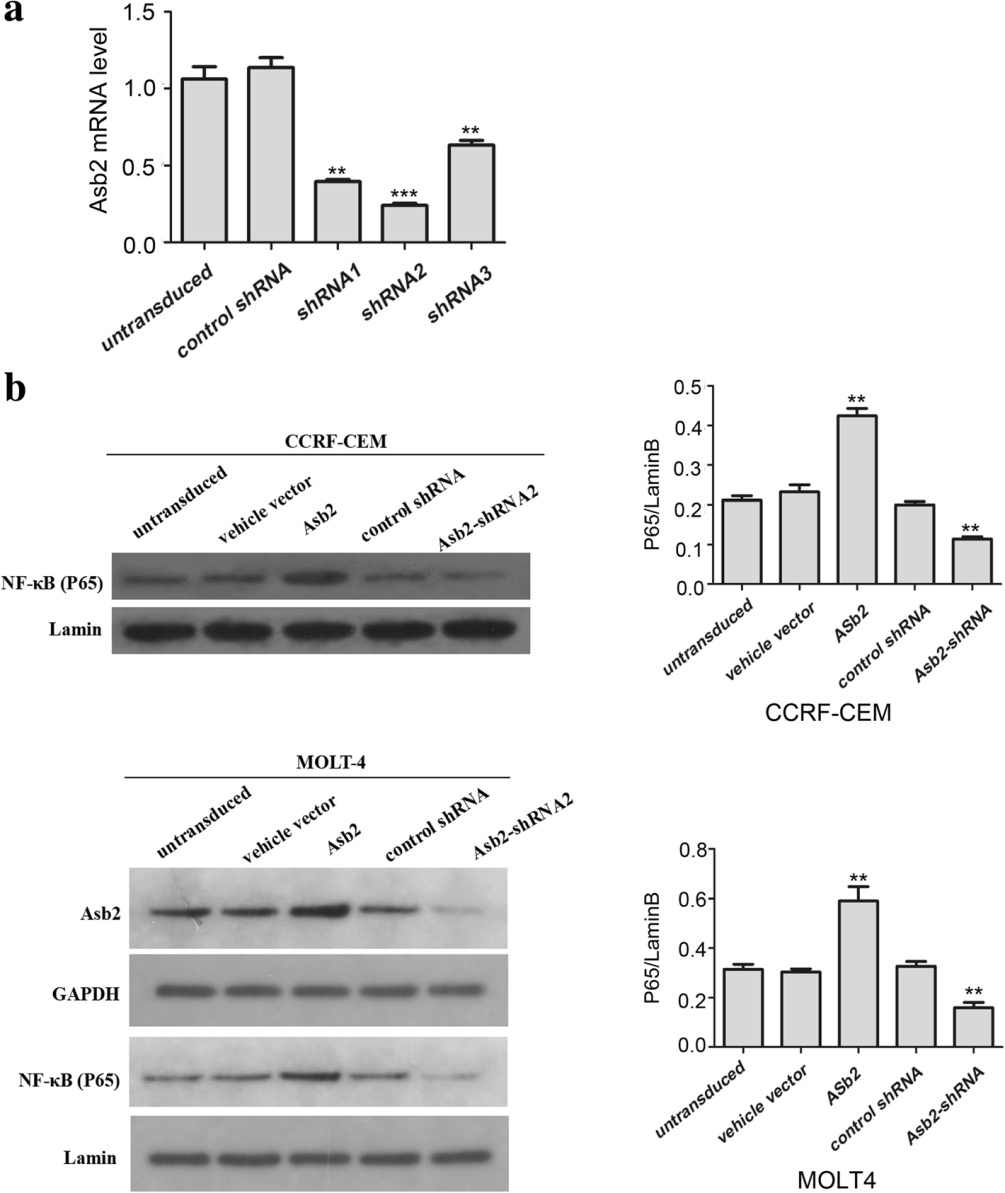
Asb2 expression promotes NF-κB activation. **a** mRNA levels of *Asb2* in CCRF-CEM cells transduced with control or *Asb2*-shRNA lentivirus. .**P < 0.01 versus control shRNA. ***P < 0.001 versus control shRNA. **b** CCRF-CEM cells and MOLT-4 cells transduced with lentivirus carrying vehicle vector, *Asb2*, control shRNA or *Asb2*-shRNA2 were analyzed for their level of NF-κB-p65 in the nucleus. **P < 0.01 versus empty vector control. The data are presented as the means ± SD. Each assay was conducted in triplicate

Because Notch signaling can up-regulate *Asb2* transcription and NF-κB activation in T-ALL cells, we investigated whether a relationship between NF-κB activation and Asb2 expression exists. To test this possibility, 3 *Asb2*-shRNAs, *Asb2*-shRNA1, *Asb2*-shRNA2 and *Asb2*-shRNA3, were created and then evaluated for their effectiveness (Fig. 2a). The results showed that all of these *Asb2*-shRNAs could significantly decrease the mRNA level of *Asb*2. Because *Asb2*-shRNA2 was the most efficient shRNA, CCRF-CEM cells and MOLT-4 cells were transduced with lentiviruses harboring wild-type *Asb2*, *Asb2*-shRNA2 or empty vector. Compared to the control, the over-expression of wild-type *Asb2* enhanced NF-κB activation, whereas knockdown of Asb2 significantly decreased NF-κB activation (Fig. 2b). These results suggest that Notch signaling regulates NF-κB activation by activating *Asb2* transcription.

### Notch signaling stimulates NF-κB activation through Asb2-induced IκBα degradation

**Fig. 3:**
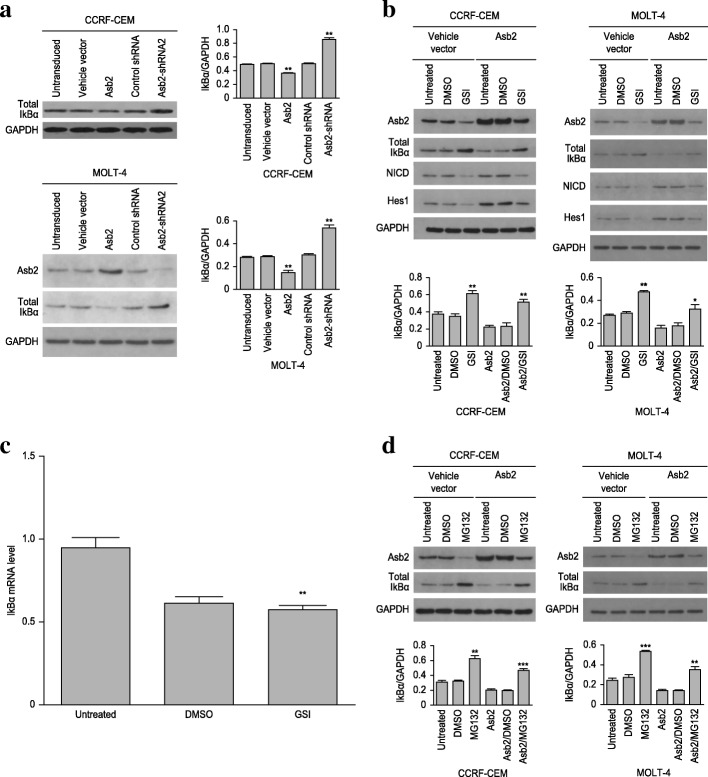
Notch signaling induces IκBα degradation through Asb2. **a** CCRF-CEM cells and MOLT-4 cells transduced with lentivirus carrying vehicle vector, *Asb2*, control shRNA or *Asb2*-shRNA2 were analyzed for their level of total IκBα. Untransduced cells served as the control. The levels of IκBα protein were then analyzed. **P < 0.01 versus empty vector control. CCRF-CEM cells and MOLT-4 cells were treated with DMSO (vehicle) or 10 μM GSI for 24 h. The total protein **b** and mRNA **c** levels of IκBα were then analyzed. Untreated cells served as the control. **P < 0.01 and *P < 0.05 versus DMSO control. **d** CCRF-CEM cells and MOLT-4 cells were treated with DMSO (vehicle) or 10 μM MG132 for 24 h. Untreated cells served as the control. The total protein levels of IκBα were then analyzed. **P < 0.01 and ***P < 0.001 versus DMSO control. The data are presented as the means ± SD. Each assay was conducted in triplicate

Given that Asb2 regulates some signaling pathways by inducing the degradation of certain proteins, Asb2 likely promotes the degradation of the NF-κB inhibitor IκBα to release and thus activate NF-κB. To test this hypothesis, CCRF-CEM cells and MOLT-4 cells were transduced with lentiviruses carrying wild-type *Asb2*, *Asb2*-shRNA or empty vector. This experiment demonstrated that the expression of exogenous *Asb2* could decrease the protein level of IκBα, whereas knockdown of Asb2 could increase the amount of IκBα (Fig. 3a). These results indicate that Asb2 can induce IκBα degradation and that Notch signaling, as an upstream regulator of Asb2, may initiate NF-κB activation by indirectly modulating IκBα degradation. To verify this conclusion, CCRF-CEM cells and MOLT-4 cells were treated with GSI or vehicle (DMSO). Indeed, inhibition of the Notch signaling pathway restored the protein level of IκBα (Fig. 3b). To rule out the possibility that the constitutively active Notch signaling pathway influences the protein level of IκBα by inhibiting IκBα transcription, CCRF-CEM cells were treated with GSI, and the mRNA levels of IκBα were then measured. The results showed that blockade of Notch signaling did not increase IκBα transcription (Fig. 3c).

### Asb2-induced IκBα degradation is proteasome dependent

Because Asb2 is known to mediate protein degradation by forming an ECS (Elongin B/C-Cul2/5-SOCS-box protein) E3 ubiquitin ligase complex, Asb2-induced IκBα degradation may be proteasome dependent. To verify this hypothesis, CCRF-CEM cells and MOLT-4 cells were treated with the proteasome inhibitor MG132. Proteasome inhibition significantly restored the protein level of IκBα in CCRF-CEM cells and MOLT-4 cells (Fig. 3d), suggesting that Asb2-induced IκBα degradation might depend on the proteasome.

### Both the SOCS box and the region linking the SOCS box to the ankyrin repeats are needed for efficient Asb2 binding

**Fig. 4:**
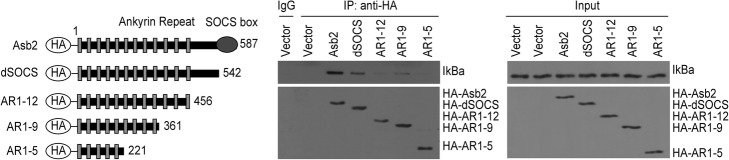
Mapping the Asb2-interacting domains in IκBα. **a** Schematic diagram of HA-tagged Asb2 and its mutant constructs. The first and last Asb2 amino acids retained in the mutant constructs are denoted. **b** The Asb2 deletion constructs described in panel A were transfected into 293 cells. Immunoprecipitation was performed with anti-HA. The precipitates and inputs were probed with anti-HA and anti-IκBα

To test whether IκBα physically interacts with Asb2, 293 cells were transfected with HA-tagged Asb2 or with empty vector. A co-immunoprecipitation (co-IP) assay was then performed using an antibody directed against the HA tag. This experiment showed that IκBα was pulled down by HA-tagged full-length Asb2 (Fig. 4), suggesting that IκBα can physically bind to Asb2 and that IκBα is a substrate of Asb2. Asb2 is composed of a SOCS box and ankyrin repeats [16, 17]. Previous studies have indicated that the SOCS box of Asb2 is responsible for interacting with the Elongin BC complex, whereas the ankyrin repeats interact with the substrate of Asb2 [16, 21]. However, one study also demonstrated that Asb2 interacts with Jak2 via sequences outside the ankyrin repeats [14]. Therefore, we asked which region of Asb2 mediates the interaction between Asb2 and IκBα. To answer this question, 293 cells were transfected with HA-tagged full-length Asb2, HA-tagged Asb2 mutants or an empty vector. A co-IP assay was then performed using an antibody directed against the HA tag. The deletion of amino acids 456–587 led to disruption of the interaction between IκBα and Asb2 (Fig. 4). A more robust interaction was observed when the SOCS box or the region linking the SOCS box to the ankyrin repeats was included (Fig. 4, compare lanes 3, 4 and 5), suggesting that both the SOCS box and the region linking the SOCS box to the ankyrin repeats are needed for efficient Asb2 binding.

### Asb2 expression promotes tumorigenesis of T-ALL cells

**Fig. 5:**
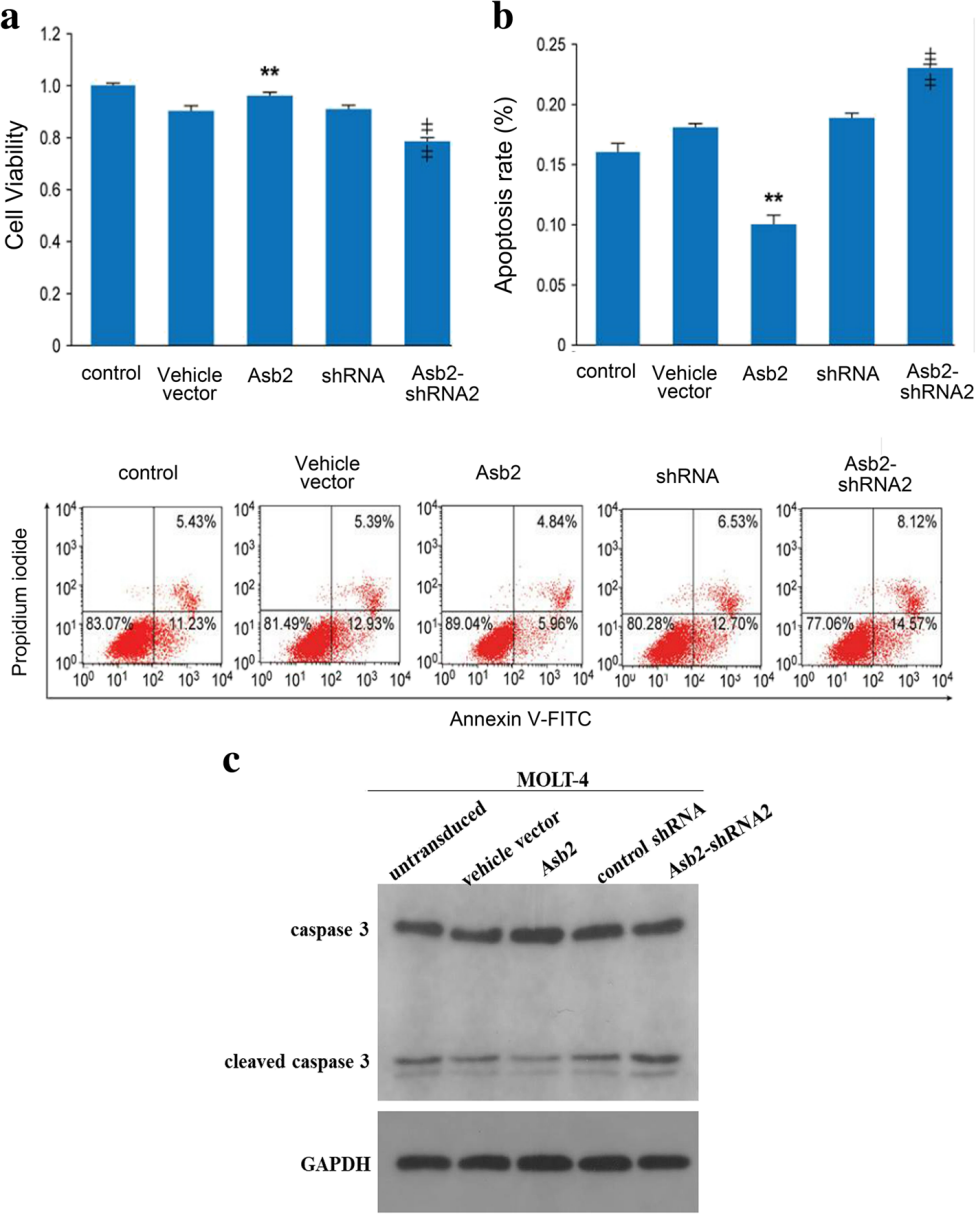
*Asb2* expression enhances viability and inhibits apoptosis of T-ALL cells. CCRF-CEM cells were transduced with lentivirus carrying empty vector, *Asb2*, control shRNA or *Asb2*-shRNA2. **a** Cell viability was analyzed by MTT assay. Each bar represents the mean ± SD of three independent experiments. **p < 0.01 versus empty vector-transduced cells; ǂ ǂ*p* < 0.01 versus control shRNA-transduced cells. Control group refers to non-transduced cells. **b** Annexin V-FITC/PI staining of the indicated cells. Each bar represents the mean ± SD of three independent experiments. **p < 0.01 versus vehicle vector-transduced cells; ǂ ǂ*p* < 0.01 versus control shRNA-transduced cells. **c** MOLT-4 cells were transduced with lentivirus carrying empty vector, *Asb2*, control shRNA or *Asb2*-shRNA2. The effects of Asb2 on apoptosis associative protein (caspase 3) in MOLT-4 were determined by Western blot with GAPDH as a loading control

Enhanced activation of NF-κB results in the occurrence of cancer by promoting cellular proliferation and inhibiting apoptosis [22]. To test whether blocking *Asb2* expression can suppress the tumorigenesis of T-ALL cells, CCRF-CEM cells and MOLT-4 cells were transduced with lentiviruses containing wild-type *Asb2*, *Asb2*-shRNA or empty vector. MTT assay results demonstrated that the expression of wild-type *Asb2* could significantly increase the viability of CCRF-CEM cells, whereas *Asb2* knockdown could decrease the viability of CCRF-CEM cells (Fig. 5a). In addition, the expression of wild-type *Asb2* led to a lower number of apoptotic CCRF-CEM cells, whereas *Asb2* knockdown resulted in an elevated number of apoptotic CCRF-CEM cells through annexin V binding (Fig. 5b). Consistent with this result, the expression of wild-type Asb2 significantly decreased the expression of cleaved caspase-3, whereas *Asb2* knockdown resulted in elevated expression of cleaved caspase-3 in MOLT-4 cells (Fig. 5c). These results suggest that Asb2 may be a key regulator whose abnormal expression can cause T-ALL.

## Discussion

A previous study found that Notch stimulates NF-κB activation by initiating the transcription of *Hes1*, which then suppresses the expression of *CYLD*, a negative regulator of IKK activity in T-ALL cells [23]; however, this finding does not rule out the possibility that other mechanisms co-exist. Our studies identified a novel mechanism for Notch-induced NF-κB activation in T-ALL cells. We first showed that Asb2 is a critical mediator of Notch-induced NF-κB activation because it targets IκBα for degradation.

Asb2 is expressed during all-trans retinoic acid-induced differentiation of promyelocytic cell lines [19]. Asb2α is also involved in hematopoietic differentiation [21]. Recently, Asb2α was observed to be expressed in dendritic cells and to play an important role in regulating the migration of immature dendritic cells by targeting the actin-binding protein filamin for degradation [20]. Taken together, these findings suggest that Asb2α exerts an extensive influence on hematopoiesis. However, no finding has yet indicated that Asb2 plays a role in the formation of T-ALL. Our study is the first to demonstrate that Asb2 is involved in the oncogenesis of T-ALL cells. These findings may lead to the development of new methods to inhibit oncogenic mechanisms that involve the abnormal activation of Notch and NF-κB.

As the substrate-binding subunit of the ECS ligase complex, Asb2 mediates the ubiquitination of certain substrates, such as the actin-binding protein filamin, as well as Jak2, Jak3 and E2A [14, 15, 24–26]. Through these diverse substrates, Asb2 influences a wide range of biological functions. However, many substrates of Asb2 remain to be discovered. Here, we add IκBα to the list of Asb2 substrates, thereby establishing a relationship between Asb2 and the NF-κB signaling pathway. The status of IκBα as an Asb2 substrate indicates that Asb2 may influence specific biological activities by regulating the NF-κB signaling pathway.

Asb2 belongs to a large protein family that has 18 members in humans [27]. Each of the Asb proteins has a SOCS box at its C-terminus and a variable number of ankyrin repeats at its N-terminus. Therefore, a certain degree of functional redundancy may exist among Asb family members. For example, a previous study showed that Asb1 behaves similarly to Asb2 in promoting E47 and Jak2 degradation [14]. Based on the findings of this study, we suspect that Asb1 may also be able to promote IκBα degradation in T-ALL cells. Thus, Asb1-induced IκBα degradation may be another mechanism that leads to the abnormal activation of NF-κB in T-ALL cells.

## Conclusion

We demonstrated that Notch1 is able to up-regulate the expression of Asb2α and activate NF-κB in T-ALL cells. Furthermore, we found that Notch1 regulates the NF-κB pathway through Asb2α, which is capable of interacting with IκBα and then inducing degradation of IκBα. We also showed that suppression of Asb2α expression can promote apoptosis and inhibit proliferation of T-ALL cells, suggesting that Asb2α may play an important role in the pathogenesis of T-ALL. Taken together, our findings might provide a promising option for targeted therapy against T-ALL.

## Abbreviations

Asb2: Ankyrin repeat and SOCS box protein 2
IκBα: Nuclear factor of kappa light polypeptide gene enhancer in B-cells inhibitor, alpha
NF-κB: Nuclear factor kappa-light-chain-enhancer of activated B cells
T-ALL: T cell acute lymphoblastic leukemia
